# Survival of mineral-bound peptides into the Miocene

**DOI:** 10.7554/eLife.82849

**Published:** 2022-12-19

**Authors:** Beatrice Demarchi, Meaghan Mackie, Zhiheng Li, Tao Deng, Matthew J Collins, Julia Clarke

**Affiliations:** 1 Department of Life Sciences and Systems Biology, University of Turin Torino Italy; 2 The Globe Institute, Faculty of Health and Medical Sciences, University of Copenhagen Copenhagen Denmark; 3 The Novo Nordisk Foundation Center for Protein Research, Faculty of Health and Medical Sciences, University of Copenhagen Copenhagen Denmark; 4 Institute of Vertebrate Paleontology and Paleoanthropology, Chinese Academy of Sciences Beijing China; 5 McDonald Institute for Archaeological Research, University of Cambridge Cambridge United Kingdom; 6 Department of Geological Sciences, The University of Texas at Austin Austin United States; University of Cape Town South Africa; Pennsylvania State University United States

**Keywords:** Struthio, eggshell, proteomics, miocene, Other

## Abstract

Previously, we showed that authentic peptide sequences could be obtained from 3.8-Ma-old ostrich eggshell (OES) from the site of Laetoli, Tanzania (Demarchi et al., 2016). Here, we show that the same sequences survive in a >6.5 Ma OES recovered from a palaeosteppe setting in northwestern China. The eggshell is thicker than those observed in extant species and consistent with the Liushu *Struthio* sp. ootaxon. These findings push the preservation of ancient proteins back to the Miocene and highlight their potential for paleontology, paleoecology, and evolutionary biology.

## Introduction

The oldest authenticated peptide sequences to date were reported in 2016 from 3.8-Ma-old ostrich eggshell (OES) from the site of Laetoli, Tanzania (Demarchi et al., 2016). This finding had great scientific impact since it integrated computational chemistry (molecular dynamics simulations) as well as experimental data to propose a mechanism of preservation, concluding that mineral binding ensures the survival of protein sequences. Importantly, this study demonstrated that peptide-bound amino acids could survive into deep time even in hot environments. The effect of temperature on the kinetics of protein diagenesis has been described by several authors, both on the basis of actualistic experiments and of the quantification of the extent of degradation in ancient samples of known ages (Crisp et al., 2013; Demarchi et al., 2013; Hendy et al., 2012; Johnson et al., 1997; Kaufman, 2006; Kaufman, 2003; Schroeder and Bada, 1976; Wehmiller, 2013; Wehmiller, 1977; Wehmiller and Belknap, 1978). This discovery has fuelled the analysis of ancient proteins from other mineral matrices, namely tooth enamel (Cappellini et al., 2019; Welker et al., 2020; Welker et al., 2019) in order to reconstruct phylogenies.

The recovery of peptides has so far been limited to biomineral samples of Plio-Pleistocene age: our attempts at retrieving intact peptides from Cretaceous eggshell were unsuccessful, despite the fact that a genuine intracrystalline fraction of amino acids was preserved in the same sample (Saitta et al., 2020). Here, we show that the same peptide sequences that exhibit strong binding to the calcite surface—and that were recovered from the Laetoli OES—also persist into the late Miocene, in a >6.5-Ma-old eggshell sample from the Linxia Basin, northeastern Tibetan Plateau, China, Liushu Formation.

## Results and discussion

**Figure 1.**
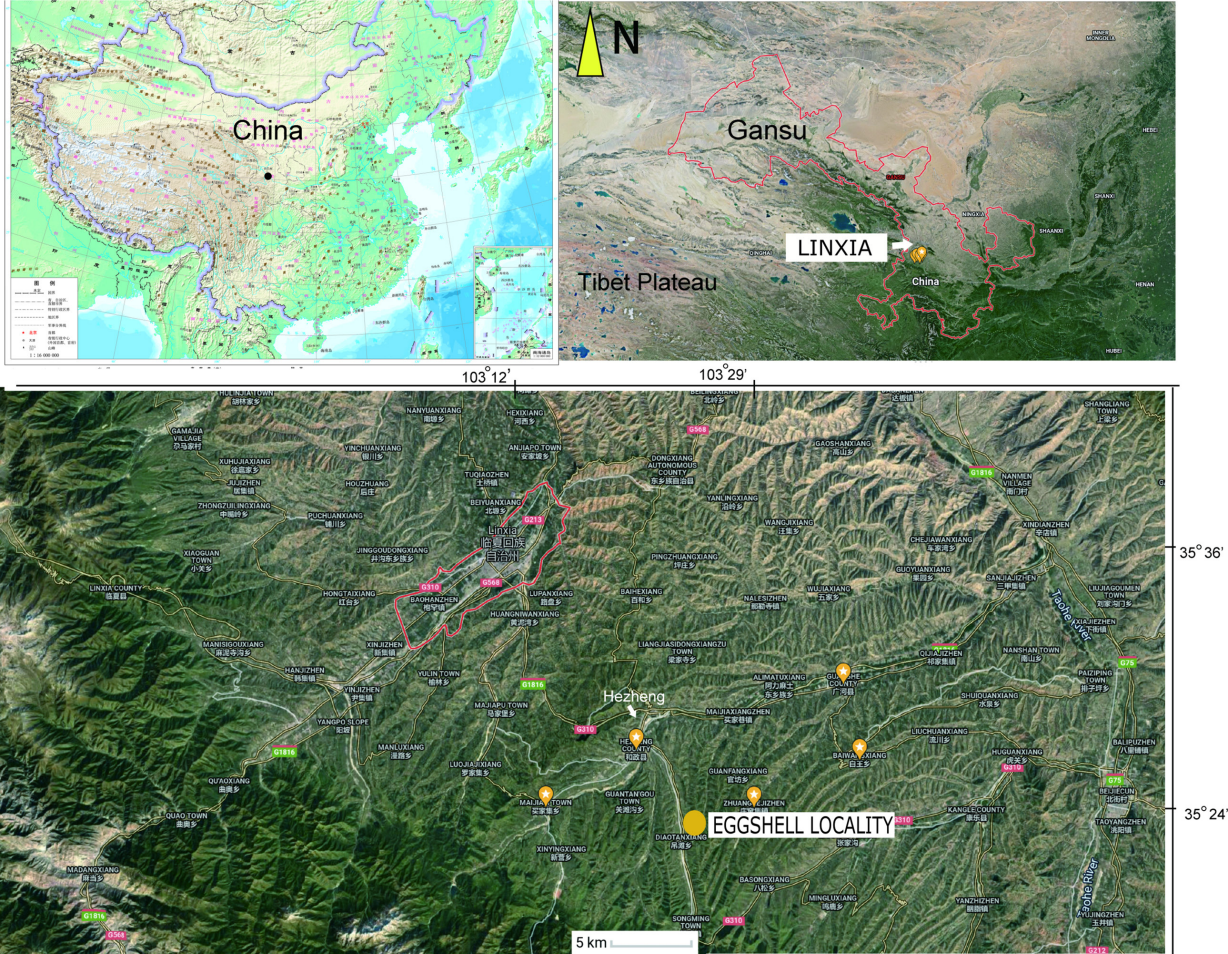
Map showing the location of the fossil eggshell site.

The OES (IVPP V26107) was recovered from an area between the towns of Xinji and Songming, in Hezheng County, close to the border with Guanghe County, from mudstone facies of the Liushu Formation (Figure 1).

Both eggshell and skeletal remains are previously known from the Late Miocene Liushu Formation, Linxia Basin, of Gansu Province, where the mean annual temperature is ∼11^◦^C (Hou et al., 2005; Liu et al., 2016; Li et al., 2021; Wang, 2008). Age control on the highly fossiliferous units of the Lishu Formation is good (Deng et al., 2019; Deng et al., 2013; Zhang et al., 2012), with detailed correlations across China as well as with Neogene deposits globally. These units have been assessed via new magnetostratigraphic data; most of the *Hipparion* fauna is estimated to be earlier than 6.4–6.5 Ma, with a lower Liushu transition to a *Platybelodon* fauna yielding age estimates of 11.1–12.5 Ma (Zhang et al., 2012). These dates agree with the prior estimates from biostratigraphic and magnetostratigraphic data (Deng et al., 2019; Deng et al., 2013). The recovery site is closer to the southern part of the basin near the Heilinding section of Zhang et al., 2012, which had an estimated minimum age of 6.4–6.5 Ma (Chron 3An). In contrast, the candidate stratotype section, Guoniguo, that exposes lower parts of the Liushu, is north of the recovery area (Deng et al., 2019; Zhang et al., 2012).

**Figure 2.**
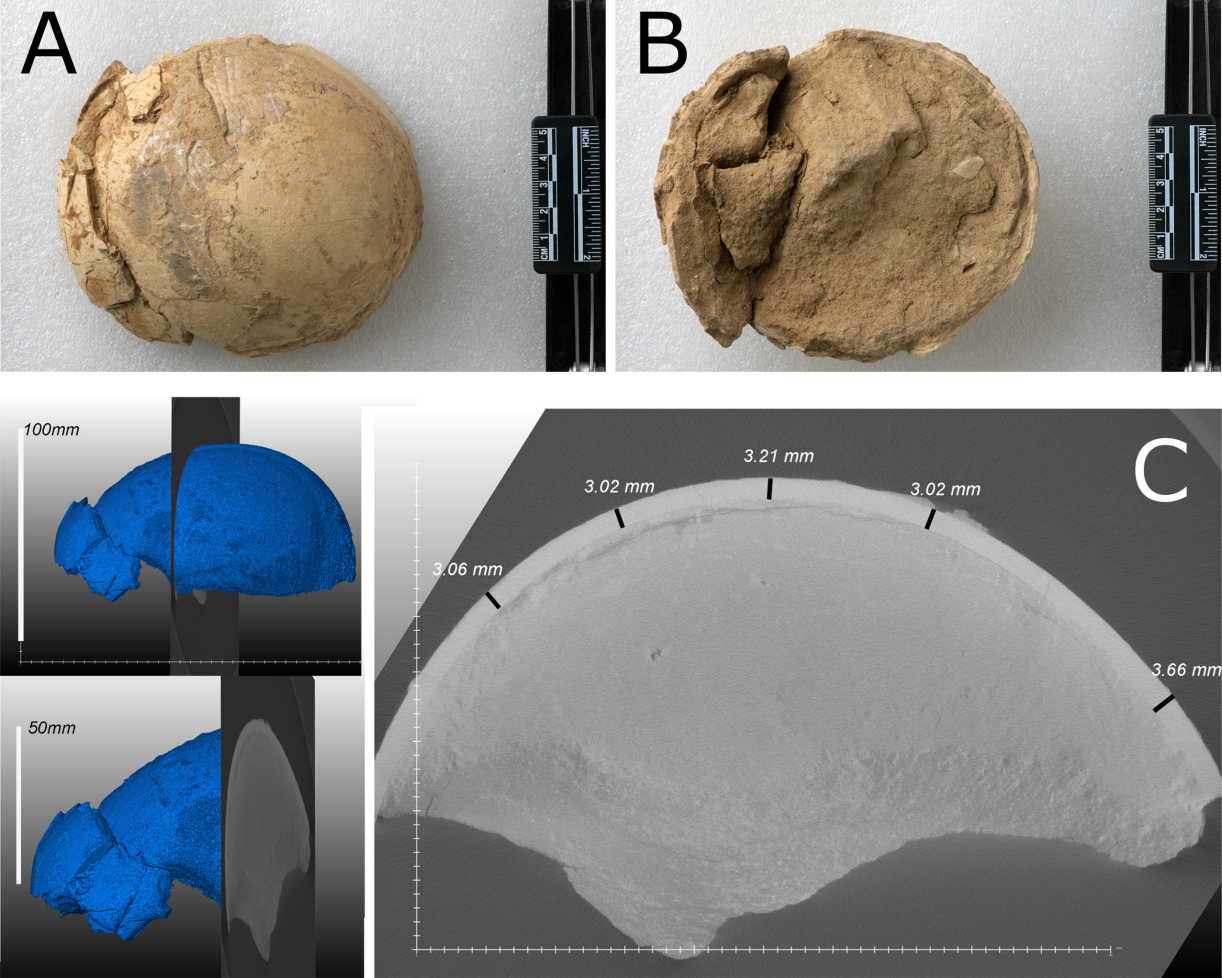
Eggshell specimen (IVPP V26107): photographs (**A, B**) and CT scan (**C**).

Ostrich (*Struthio*) remains to have a long history of recovery from the late Miocene of northwest China (Buffetaut and Angst, 2021; Hou et al., 2005; Li et al., 2021; Lowe, 1931; Mikhailov and Zelenkov, 2020). The first reported ostrich fossils from China were from units of the ‘*Hipparion* Clay’ (Red Clay) dated between 6.54 and 7.18 Ma (Lowe, 1931; Zhu et al., 2008). Mikhailov and Zelenkov, 2020 concluded that the Wang, 2008 Liushu taxon is referable to *Struthio* and not presently supported as a distinct but related genus; it is proposed to be from a taxon larger than extant *Struthio* species with a thickness of ~2.4 mm consistent with the eggshell sampled here (Figure 2C).

**Figure 3.**
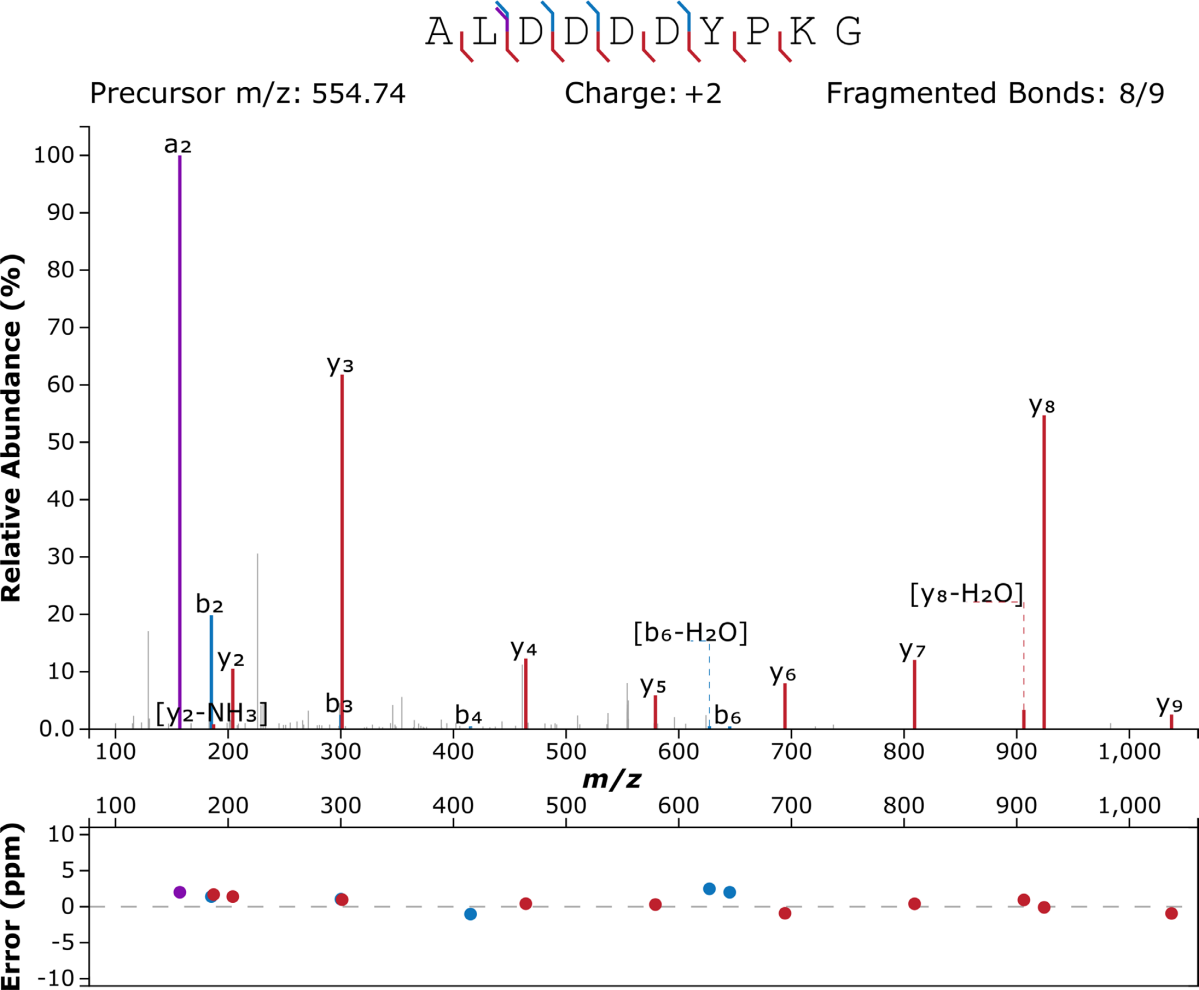
Annotated product ion spectrum of peptide ALDDDDYPKG, m/z 554.749, −10lgP=32.57. Figure created using http://www.interactivepeptidespectralannotator.com/PeptideAnnotator.html (Brademan et al., 2019).

**Figure 4.**
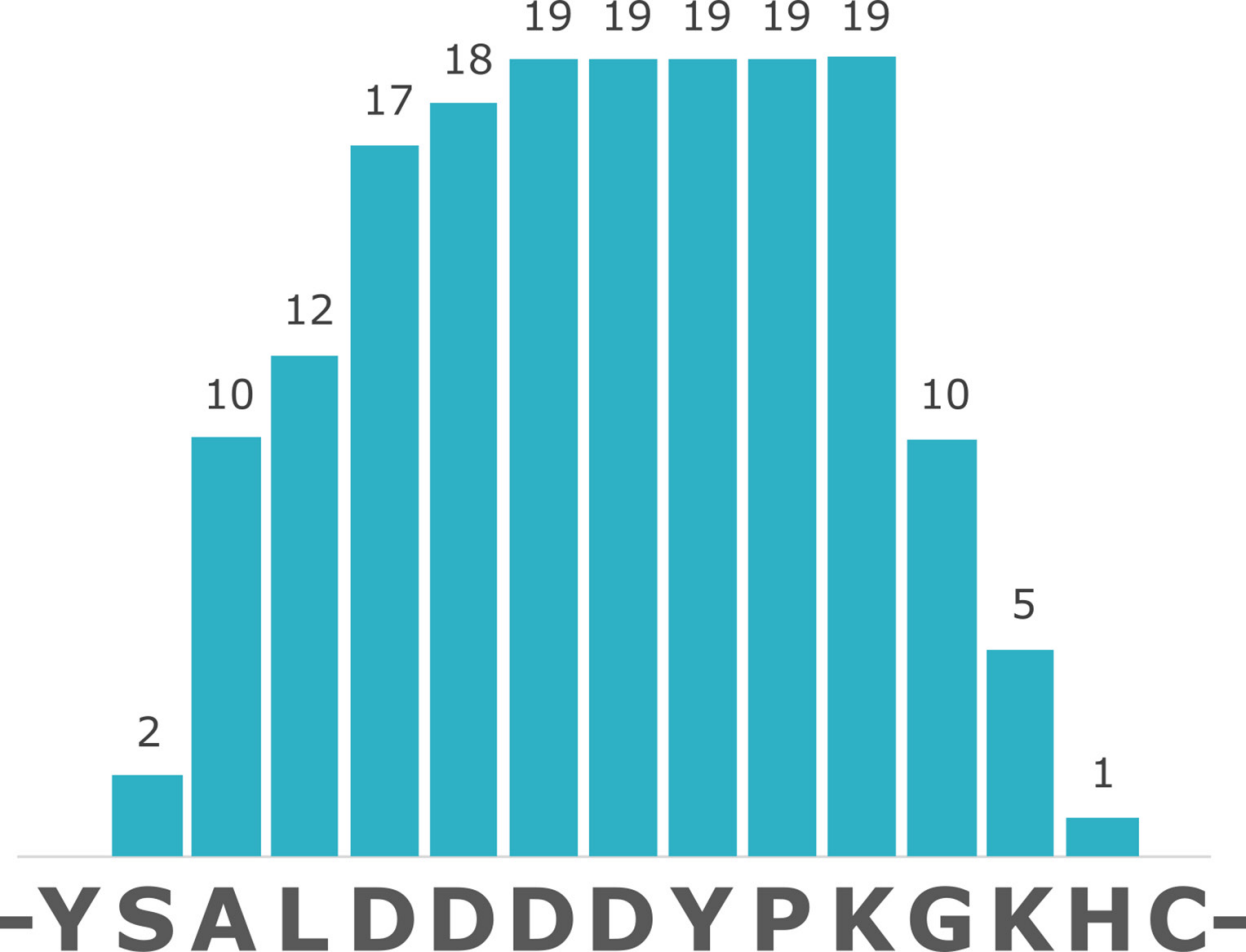
Coverage of struthiocalcin-1 (SCA-1) in Miocene OES specimen IVPP V26107. Numbers indicate spectrum matches for each position in the sequence.

Given the extreme antiquity of the samples, protein extraction was performed in an ultra-clean facility at the University of Copenhagen in order to minimize any chance of contamination, following Hendy et al., 2018. Furthermore, OES powders were bleached extensively to isolate the intracrystalline fraction only (as described by Demarchi et al., 2016), samples were analysed by LC-MS/MS using a new LC column, and six blanks (analytical and procedural) were included in the run, flanking the sample. Data analysis was equally cautious: raw tandem mass spectrometry data were used to reconstruct potential peptide sequences starting from raw product ion spectra; only de novo peptides with an ALC (Average Local Confidence) score ≥80% were considered. This is the most stringent threshold that can be applied, and it signifies that all potential peptide sequences with lower confidence are discarded. The de novo peptides were searched against the Uniprot/Swissprot database (containing 565,254 manually annotated and reviewed protein sequences). A database of common laboratory contaminants (common Repository of Adventitious Protein [cRAP]) was included in the search. Eleven unique peptide sequences were found to match the sequence of struthiocalcin-1 (Table 1), all containing the typical Asp-rich motif ‘DDDD’ (Figures 3 and 4), which had been shown in our previous paper to be the mineral-binding peptide belonging to the sequence of struthiocalcin-1 (SCA-1). Annotated tandem mass spectra are shown in Figure 3 (ALDDDDYPKG) and Figure 3—figure supplement 1 (ALDDDYPK), Figure 3—figure supplement 2 (SALDDDDYPKG), Figure 3—figure supplement 3 (DDDDYPKGKH), Figure 3—figure supplement 4 (LDDDDYPKGK), Figure 3—figure supplement 5 (SALDDDDYPK), Figure 3—figure supplement 6 (DDDDYPKGK), Figure 3—figure supplement 7 (LDDDDYPKG), Figure 3—figure supplement 8 (DDDYPKGK), Figure 3—figure supplement 9 (DDDDYPK), and Figure 3—figure supplement 10 (DDYPKGK).

**Table 1.** Peptide sequences identified in sample IVPP V26107.

Scan	Precursor mass	z	*m/z*	Peptide sequence	Peptide mass	Error (ppm)	Peptide –10lgP
5401	1107.472	2	555.246	ALDDDDYPKG	1107.472	5.7	27.24
5268	1050.457	2	526.236	ALDDDDYPK	1050.451	6.1	24.83
5051	1194.517	2	598.266	SALDDDDYPKG	1194.504	10.9	21.11
4984	1137.494	2	569.754	SALDDDDYPK	1137.483	10.1	29.94
4727	1137.494	2	569.755	SALDDDDYPK	1137.483	9.7	16.76
4565	1107.483	2	554.749	ALDDDDYPKG	1107.472	9.8	32.57
4565	1107.484	2	554.749	ALDDDDYPKG	1107.472	10.6	28.7
4443	1050.459	2	526.237	ALDDDDYPK	1050.451	7.6	17.62
4313	1050.461	2	526.235	ALDDDDYPK	1050.451	9.9	15.13
4199	1050.458	2	526.236	ALDDDDYPK	1050.451	7.2	16.35
4037	1036.442	2	519.230	LDDDDYPKG	1036.435	7.2	16.57
2724	1164.539	2	583.277	LDDDDYPKGK	1164.53	8.2	30.91
2502	866.335	2	434.176	DDDDYPK	866.335	6.9	18.46
2176	866.335	2	434.175	DDDDYPK	866.335	7.1	21.18
2026	866.338	2	434.176	DDDDYPK	866.329	10.1	19.07
1396	1051.454	2	526.734	DDDDYPKGK	1051.446	7.8	28.62
1138	936.427	2	469.221	DDDYPKGK	936.419	8.4	27.8
1046	1188.513	3	397.178	DDDDYPKGKH	1188.505	7.2	30.65
1042	821.398	2	411.706	DDYPKGK	821.392	7.6	17.39

**Figure 3—figure supplement 1.**
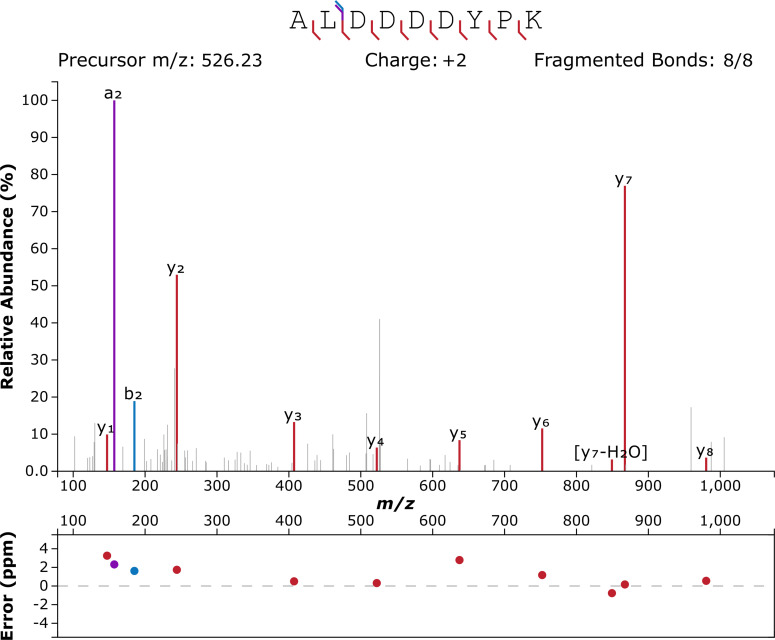
Annotated product ion spectrum, ALDDDDYPK, m/z 526.236, –10lgP=24.83. Figure created using http://www.interactivepeptidespectralannotator.com/PeptideAnnotator.html (Brademan et al., 2019).

**Figure 3—figure supplement 2.**
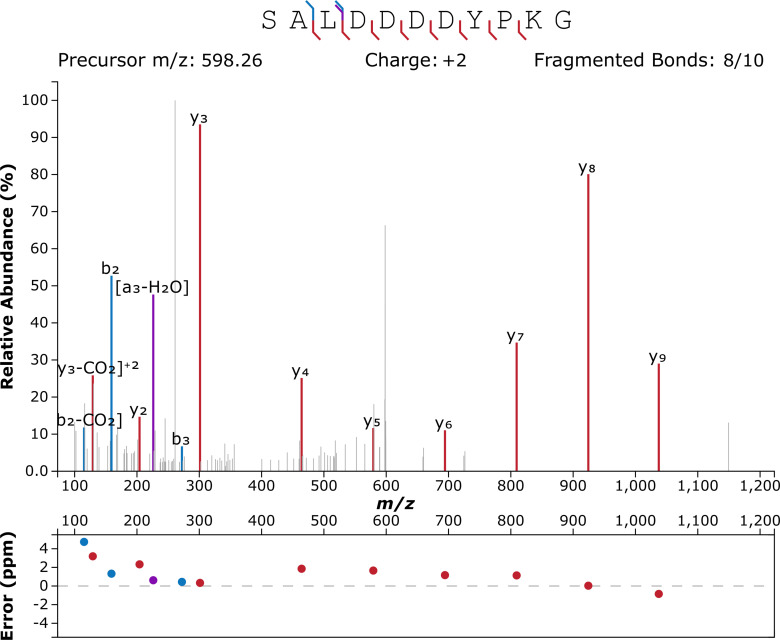
Annotated product ion spectrum, SALDDDDYPKG, m/z 598.266, −10lgP=21.11. Figure created using http://www.interactivepeptidespectralannotator.com/PeptideAnnotator.html (Brademan et al., 2019).

**Figure 3—figure supplement 3.**
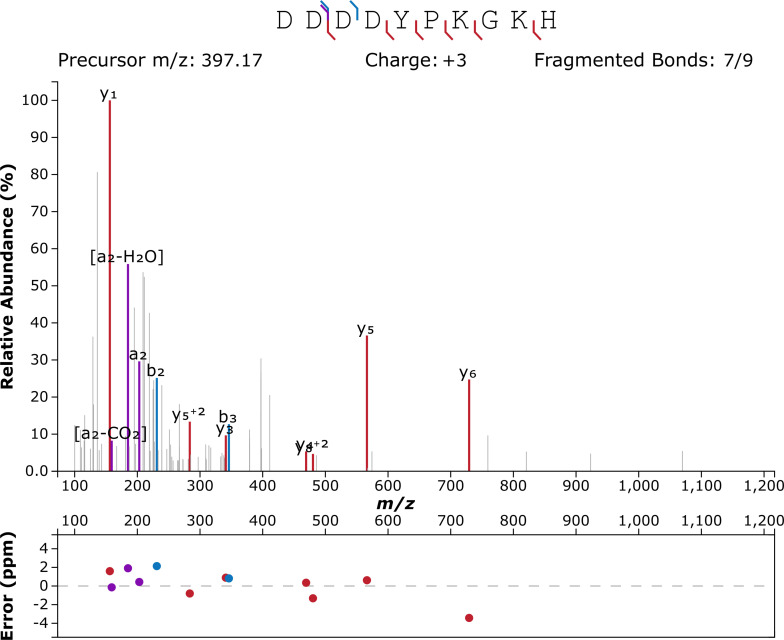
Annotated product ion spectrum, DDDDYPKGKH, m/z 397.178, −10lgP=30.65. Figure created using http://www.interactivepeptidespectralannotator.com/PeptideAnnotator.html (Brademan et al., 2019).

**Figure 3—figure supplement 4.**
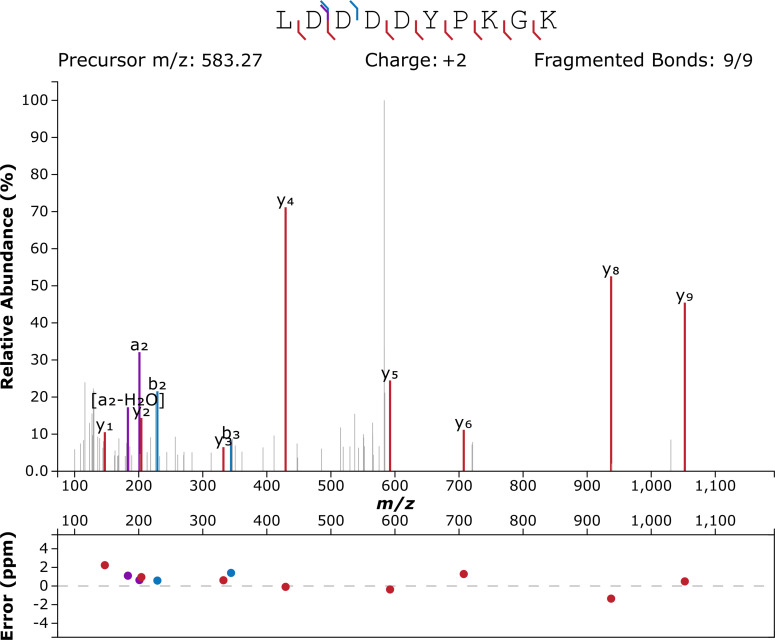
Annotated product ion spectrum, LDDDDYPKGK, m/z 583.277, −10lgP=30.91. Figure created using http://www.interactivepeptidespectralannotator.com/PeptideAnnotator.html (Brademan et al., 2019).

**Figure 3—figure supplement 5.**
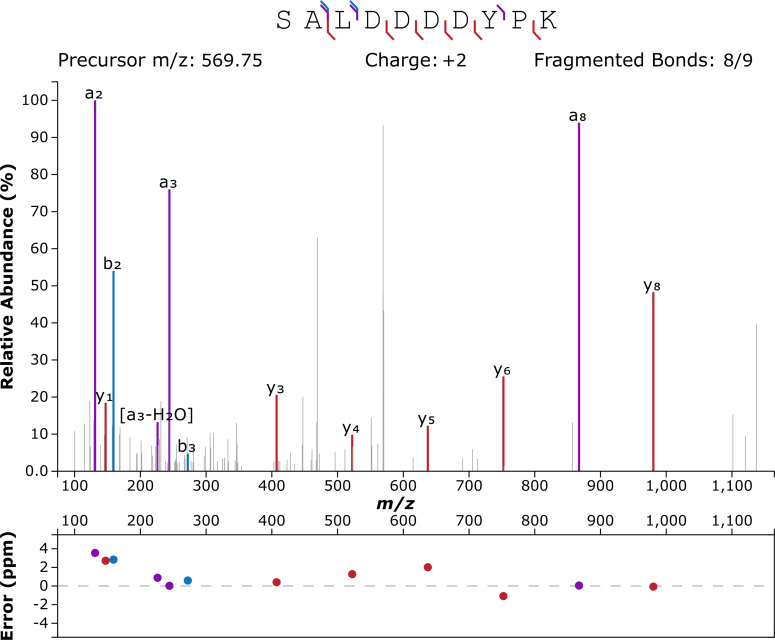
Annotated product ion spectrum, SALDDDDYPK, m/z 569.754, −10lgP=29.94. Figure created using http://www.interactivepeptidespectralannotator.com/PeptideAnnotator.html (Brademan et al., 2019).

**Figure 3—figure supplement 6.**
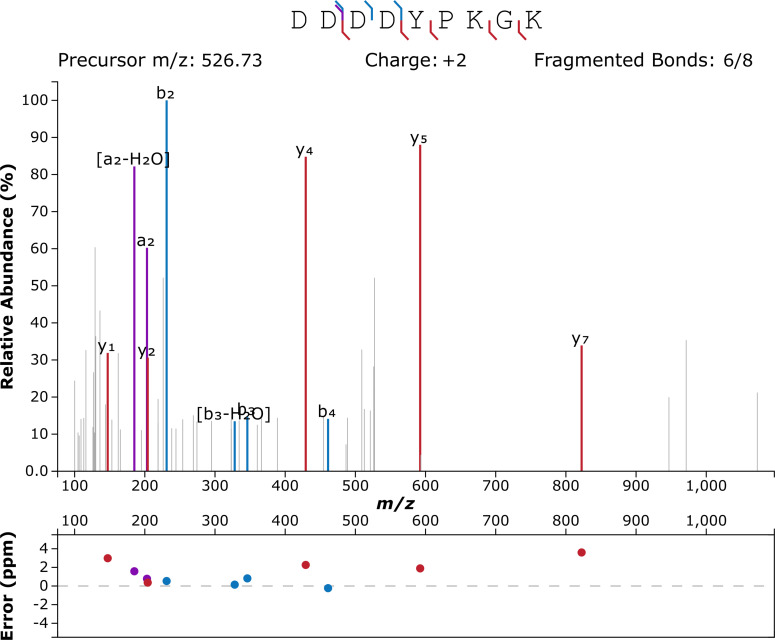
Annotated product ion spectrum, DDDDYPKGK, m/z=526.734, −10lgP=28.62. Figure created using http://www.interactivepeptidespectralannotator.com/PeptideAnnotator.html (Brademan et al., 2019).

**Figure 3—figure supplement 7.**
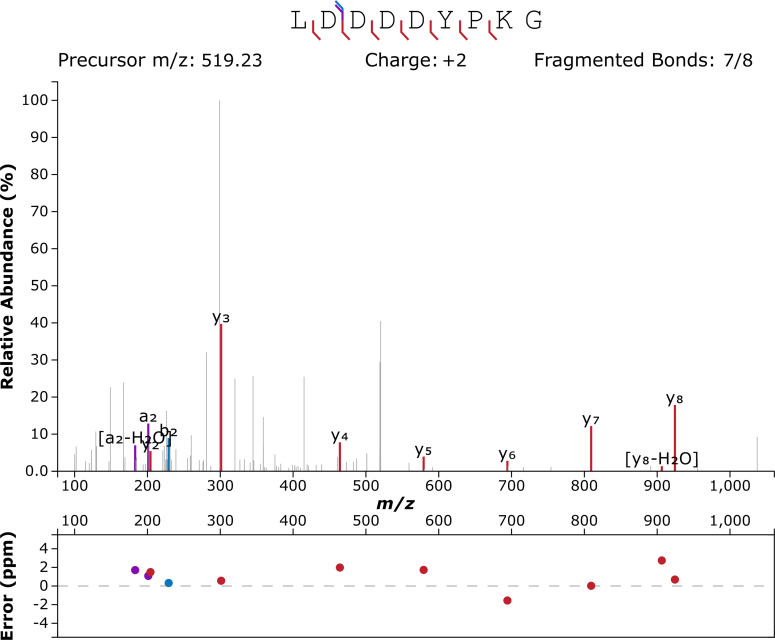
Annotated product ion spectrum, LDDDDYPKG, m/z=519.23, −10lgP=16.57. Figure created using http://www.interactivepeptidespectralannotator.com/PeptideAnnotator.html (Brademan et al., 2019).

**Figure 3—figure supplement 8.**
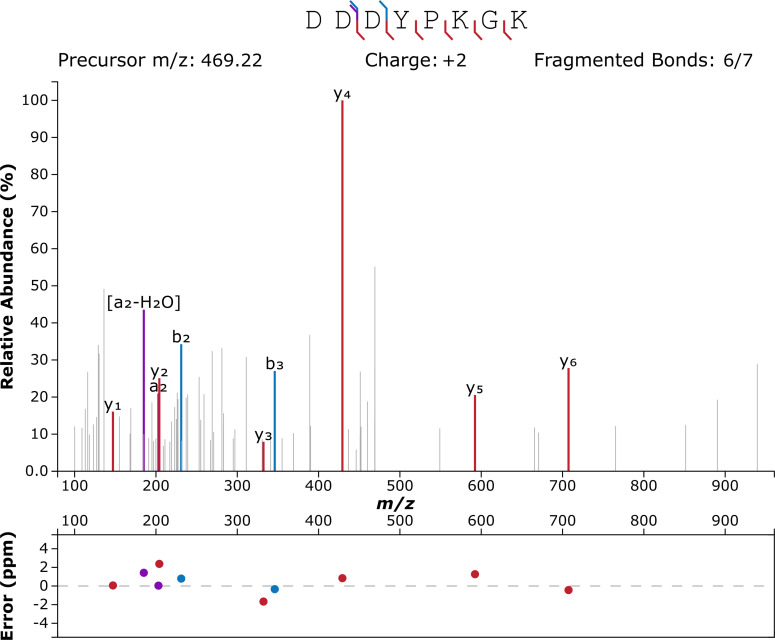
Annotated product ion spectrum, DDDYPKGK, m/z=469.221, −10lgP=27.8. Figure created using http://www.interactivepeptidespectralannotator.com/PeptideAnnotator.html (Brademan et al., 2019).

**Figure 3—figure supplement 9.**
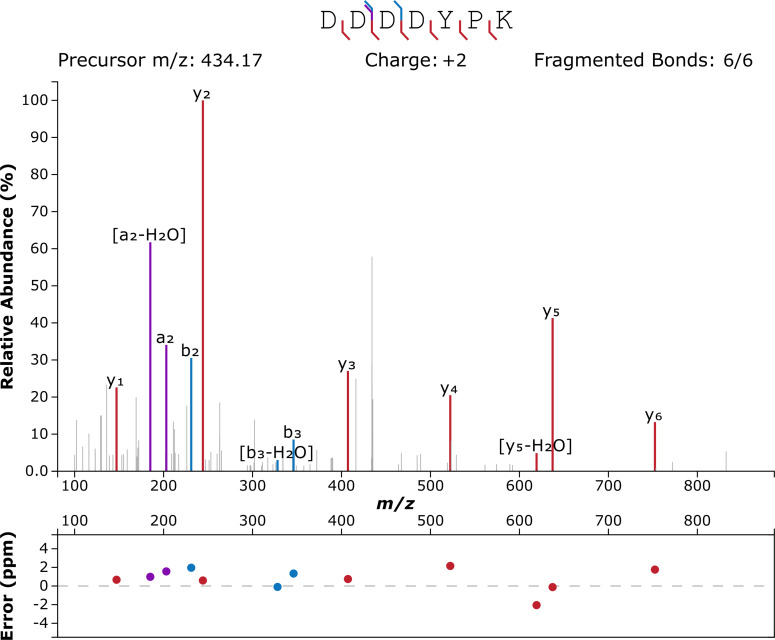
Annotated product ion spectrum, DDDDYPK, m/z=434.174, −10lgP=21.18. Figure created using http://www.interactivepeptidespectralannotator.com/PeptideAnnotator.html (Brademan et al., 2019).

**Figure 3—figure supplement 10.**
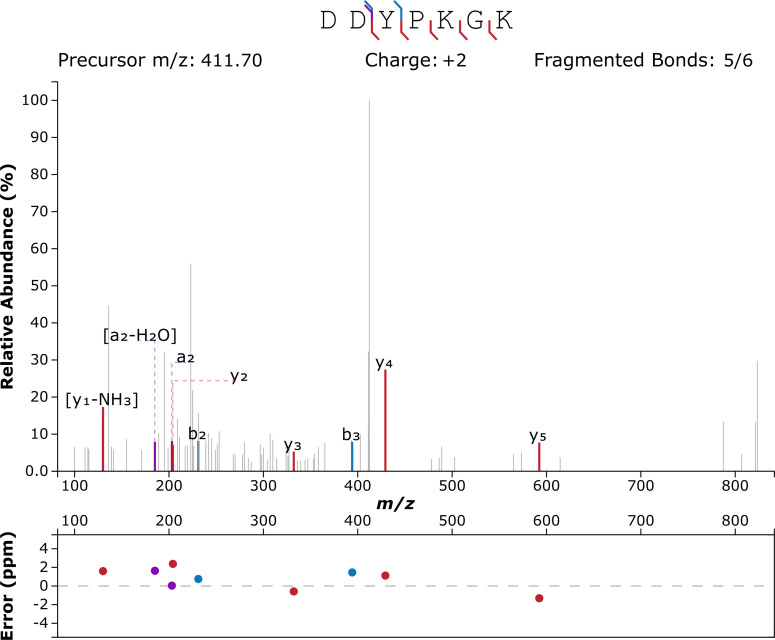
Annotated product ion spectrum, DDYPKGK, m/z=411.706, −10lgP=17.39. Figure created using http://www.interactivepeptidespectralannotator.com/PeptideAnnotator.html (Brademan et al., 2019).

### Conclusions

We present the first evidence for peptide survival into the Miocene, confirming our previous (Pliocene) data and providing further support to the mechanism of preservation based on the binding of Asx-rich peptides to calcite surfaces (Demarchi et al., 2016). The sequence recovered from the Chinese specimen is identical to the peptides found in the 3.8 Ma Laetoli OES, thus also supporting the attribution of the Liushu ootaxon to genus *Struthio*. While the four Asp residues are conserved across several avian taxa, in some species, including other ratites, Asp can be substituted by Glu and some of the flanking residues are also variable (Demarchi et al., 2022, fig. S1); therefore, variability within this sequence could be informative for evolutionary relationships between extinct and extant taxa. The absolute age of the Chinese sample is greater than that of the African material (Demarchi et al., 2016), but, given the latitudinal differences between the two sites, it is likely to have experienced lower temperatures throughout its burial history. This suggests that sequences of even greater antiquity may be recovered from biominerals harboring a closed system of proteins, particularly from sites in cold environments (e.g., high altitude and/or latitude).

## Materials and methods

### Sample preparation and analysis

A subsample of OES specimen IVPP V26107 was prepared in the ultra-clean facility at the University of Copenhagen, following the protocol of Demarchi et al., 2016; Demarchi et al., 2022 and omitting the digestion step. In brief, the fragment was powdered, bleached for 72 hr (NaOCl, 15% w/v) and demineralized in cold weak hydrochloric acid (0.6 M HCl). The acid was added in 200 µl increments until it stopped reacting, for a total volume of 1400 µl 0.6 M HCl. The extracts were exchanged in ammonium bicarbonate buffer (pH=7.8) using 3 kDa MWCO ultrafilters and the peptides were purified and concentrated using C18 Stage Tips (Cappellini et al., 2019; Rappsilber et al., 2007).

Prepared StageTips of the procedural blank and sample were eluted with 30 µl of 40% acetonitrile (ACN) 0.1% formic acid into a 96-well plate prior to LC-MS/MS analysis. To remove the ACN, the plate was vacuum centrifuged until approximately 5 µl remained. Samples were then resuspended with 6 µl of 0.1% trifluoroacetic acid (TFA) 5% ACN. Based on protein concentration results at 205 nm (NanoDrop, Thermo Fisher Scientific), 5 µl of each sample and procedural blank was then separated over a 77 min gradient by an EASY-nLC 1200 (Proxeon, Odense, Denmark) attached to a Q-Exactive HF-X mass spectrometer (Thermo Fisher Scientific, Germany) using a 15 cm column. The column (75 μm inner diameter) was made in-house, laser pulled, and packed with 1.9 μm C18 beads (Dr. Maisch, Germany). Parameters were the same as those already published for historical samples (Mackie et al., 2018). In short, MS1: 120k resolution, maximum injection time (IT) 25 ms, scan target 3E6. MS2: 60k resolution, top 10 mode, maximum IT 118 ms, minimum scan target 3E3, normalized collision energy of 28, dynamic exclusion 20 s, and isolation window of 1.2 m/z. Wash-blanks consisting of 0.1% TFA 5% ACN were also run in order to hinder cross-contamination. The LC-MS/MS run included, in this order: two wash blanks, one procedural blank, one wash blank, the OES sample, and two wash blanks. The data sets have been deposited to the ProteomeXchange Consortium via the Proteomics Identifications Database (PRIDE) partner repository with the identifier PXD035872.

### Data analysis

Bioinformatic analysis was carried out using PEAKS Studio 8.5 (Bioinformatics Solutions Inc; Zhang et al., 2012). The Uniprot_swissprot database (downloaded 11/08/2021) was used for carrying out the searches and common contaminants were included (cRAP: http://www.thegpm.org/crap/). No enzyme was specified for the digestion and the tolerance was set to 10 ppm on the precursor and 0.05 Da on the fragments. The thresholds for peptide and protein identification were set as follows: peptide score −10lgP≥15, protein score −10lgP≥20, and de novo sequences scores (ALC%)≥80.

## Data Availability

Tandem mass spectra supporting peptide sequence identification are reported in Figure 3 and Figure 3 - Supplement 1 to 10. Raw mass spectrometry data and results of bioinformatics analysis are available via ProteomeXchange with identifier PXD035872. The following dataset was generated: DemarchiB 2022Ostrich eggshell peptides survival into the Miocene (6-9 Ma)PRIDEPXD035872

## Additional files

Transparent reporting form

## References

[bib1] Brademan DR, Riley NM, Kwiecien NW, Coon JJ (2019). Interactive peptide spectral annotator: a versatile web-based tool for proteomic applications. Molecular & Cellular Proteomics.

[bib2] Buffetaut E, Angst D (2021). A giant ostrich from the Lower Pleistocene Nihewan formation of North China, with a review of the fossil ostriches of China. Diversity.

[bib3] Cappellini E, Welker F, Pandolfi L, Ramos-Madrigal J, Samodova D, Rüther PL, Fotakis AK, Lyon D, Moreno-Mayar JV, Bukhsianidze M, Rakownikow Jersie-Christensen R, Mackie M, Ginolhac A, Ferring R, Tappen M, Palkopoulou E, Dickinson MR, Stafford TW, Chan YL, Götherström A, Nathan S, Heintzman PD, Kapp JD, Kirillova I, Moodley Y, Agusti J, Kahlke RD, Kiladze G, Martínez-Navarro B, Liu S, Sandoval Velasco M, Sinding MHS, Kelstrup CD, Allentoft ME, Orlando L, Penkman K, Shapiro B, Rook L, Dalén L, Gilbert MTP, Olsen JV, Lordkipanidze D, Willerslev E (2019). Early Pleistocene enamel proteome from Dmanisi resolves *Stephanorhinus* phylogeny. Nature.

[bib4] Crisp M, Demarchi B, Collins M, Morgan-Williams M, Pilgrim E, Penkman K (2013). Isolation of the intra-crystalline proteins and kinetic studies in *Struthio camelus* (ostrich) eggshell for amino acid geochronology. Quaternary Geochronology.

[bib5] Demarchi B, Collins MJ, Tomiak PJ, Davies BJ, Penkman KEH (2013). Intra-crystalline protein diagenesis (ICPD) in *Patella vulgata* part II: breakdown and temperature sensitivity. Quaternary Geochronology.

[bib6] Demarchi B, Hall S, Roncal-Herrero T, Freeman CL, Woolley J, Crisp MK, Wilson J, Fotakis A, Fischer R, Kessler BM, Rakownikow Jersie-Christensen R, Olsen JV, Haile J, Thomas J, Marean CW, Parkington J, Presslee S, Lee-Thorp J, Ditchfield P, Hamilton JF, Ward MW, Wang CM, Shaw MD, Harrison T, Domínguez-Rodrigo M, MacPhee RDE, Kwekason A, Ecker M, Kolska Horwitz L, Chazan M, Kröger R, Thomas-Oates J, Harding JH, Cappellini E, Penkman K, Collins MJ (2016). Protein sequences bound to mineral surfaces persist into deep time. eLife.

[bib7] Demarchi B, Stiller J, Grealy A, Mackie M, Deng Y, Gilbert T, Clarke J, Legendre LJ, Boano R, Sicheritz-Pontén T, Magee J, Zhang G, Bunce M, Collins MJ, Miller G (2022). Ancient proteins resolve controversy over the identity of *Genyornis* eggshell. PNAS.

[bib8] Deng T, Qiu ZX, Wang BY, Wang X, Hou SK (2013). Late Cenozoic Biostratigraphy of the Linxia Basin, Northwestern China. Fossil Mammals of Asia.

[bib9] Deng T, Hou S, Wang S (2019). Neogene integrative stratigraphy and timescale of China. Science China Earth Sciences.

[bib10] Hendy EJ, Tomiak PJ, Collins MJ, Hellstrom J, Tudhope AW, Lough JM, Penkman KEH (2012). Assessing amino acid racemization variability in coral intra-crystalline protein for geochronological applications. Geochimica Cosmochimica Acta.

[bib11] Hendy J, Welker F, Demarchi B, Speller C, Warinner C, Collins MJ (2018). A guide to ancient protein studies. Nature Ecology & Evolution.

[bib12] Hou L, Zhou Z, Zhang F, Wang Z (2005). A Miocene ostrich fossil from Gansu province, northwest China. Chinese Science Bulletin.

[bib13] Johnson BJ, Miller GH, Fogel ML, Beaumont PB (1997). The determination of late quaternary paleoenvironments at Equus cave, South Africa, using stable isotopes and amino acid racemization in ostrich eggshell. Palaeogeography, Palaeoclimatology, Palaeoecology.

[bib14] Kaufman DS (2003). Amino acid paleothermometry of Quaternary ostracodes from the Bonneville basin, Utah. Quaternary Science Reviews.

[bib15] Kaufman DS (2006). Temperature sensitivity of aspartic and glutamic acid racemization in the foraminifera *Pulleniatina*. Quaternary Geochronology.

[bib16] Li Z, Bailleul AM, Stidham TA, Wang M, Deng T (2021). Exceptional preservation of an extinct ostrich from the Late Miocene Linxia basin of China. Vertebrata PalAsiatica.

[bib17] Liu J, Li JJ, Song CH, Yu H, Peng TJ, Hui ZC, Ye XY (2016). Palynological evidence for Late Miocene stepwise aridification on the northeastern Tibetan Plateau. Climate of the Past.

[bib18] Lowe PR (1931). Struthious remains from northern China and Mongolia: with descriptions of Struthio wimani, Struthio anderssoni and Struthio mongolicus spp. nov.

[bib19] Mackie M, Rüther P, Samodova D, Di Gianvincenzo F, Granzotto C, Lyon D, Peggie DA, Howard H, Harrison L, Jensen LJ, Olsen JV, Cappellini E (2018). Palaeoproteomic profiling of conservation layers on a 14th century italian wall painting. Angewandte Chemie.

[bib20] Mikhailov KE, Zelenkov N (2020). The Late Cenozoic history of the ostriches (Aves: Struthionidae), as revealed by fossil eggshell and bone remains. Earth-Science Reviews.

[bib21] Rappsilber J, Mann M, Ishihama Y (2007). Protocol for micro-purification, enrichment, pre-fractionation and storage of peptides for proteomics using stagetips. Nature Protocols.

[bib22] Saitta ET, Vinther J, Crisp MK, Abbott GD, Kaye TG, Pittman M, Bull I, Fletcher I, Chen X, Collins MJ, Sakalauskaite J, Mackie M, Dal Bello F, Dickinson MR, Stevenson MA, Donohoe P, Heck PR, Demarchi B, Penkman KEH (2020). Non-Avian Dinosaur Eggshell Calcite Contains Ancient, Endogenous Amino Acids. bioRxiv.

[bib23] Schroeder RA, Bada JL (1976). A review of the geochemical applications of the amino acid racemization reaction. Earth-Science Reviews.

[bib24] Wang S (2008). Reexamination of taxonomic assignment of *Struthio linxiaensis* Hou et al., 2005. Acta Palaeontol Sin.

[bib25] Wehmiller JF (1977). Amino acid studies of the Del Mar, California, midden site: apparent rate constants, ground temperature models, and chronological implications. Earth and Planetary Science Letters.

[bib26] Wehmiller JF, Belknap DF (1978). Alternative kinetic models for the interpretation of amino acid enantiomeric ratios in Pleistocene mollusks: examples from California, Washington, and Florida. Quaternary Research.

[bib27] Wehmiller JF (2013). United States quaternary coastal sequences and molluscan racemization geochronology – what have they meant for each other over the past 45 years?. Quaternary Geochronology.

[bib28] Welker F, Ramos-Madrigal J, Kuhlwilm M, Liao W, Gutenbrunner P, de Manuel M, Samodova D, Mackie M, Allentoft ME, Bacon AM, Collins MJ, Cox J, Lalueza-Fox C, Olsen JV, Demeter F, Wang W, Marques-Bonet T, Cappellini E (2019). Enamel proteome shows that *Gigantopithecus* was an early diverging pongine. Nature.

[bib29] Welker F, Ramos-Madrigal J, Gutenbrunner P, Mackie M, Tiwary S, Rakownikow Jersie-Christensen R, Chiva C, Dickinson MR, Kuhlwilm M, de Manuel M, Gelabert P, Martinón-Torres M, Margvelashvili A, Arsuaga JL, Carbonell E, Marques-Bonet T, Penkman K, Sabidó E, Cox J, Olsen JV, Lordkipanidze D, Racimo F, Lalueza-Fox C, Bermúdez de Castro JM, Willerslev E, Cappellini E (2020). The dental proteome of *Homo antecessor*. Nature.

[bib30] Zhang J, Xin L, Shan B, Chen W, Xie M, Yuen D, Zhang W, Zhang Z, Lajoie GA, Ma B (2012). PEAKS DB: de novo sequencing assisted database search for sensitive and accurate peptide identification. Molecular & Cellular Proteomics.

[bib31] Zhu Y, Zhou L, Mo D, Kaakinen A, Zhang Z, Fortelius M (2008). A new magnetostratigraphic framework for Late Neogene Hipparion Red Clay in the eastern loess plateau of China. Palaeogeography, Palaeoclimatology, Palaeoecology.

